# *“It’s about wanting to disappear from the world… ”* - an interpretative phenomenological analysis on the meaning of music and hearing-related risks

**DOI:** 10.1080/17482631.2025.2480966

**Published:** 2025-03-19

**Authors:** Iris Elmazoska, Staffan Bengtsson, Stephen Widén

**Affiliations:** a Faculty of Medicine and Health, School of Health Sciences, Örebro University, Örebro, Sweden; b Audiological Research Centre, Örebro University, Örebro, Sweden; c Department of Social Work, School of Health and Welfare, Jönköping University, Jönköping, Sweden

**Keywords:** Music, meaning, adolescent, hearing health, risk awareness, well-being, health promotion

## Abstract

**Purpose:**

To explore the role and meaning of music in adolescents’ lives and the adolescents’ ways of understanding how music listening can impact hearing-health.

**Methods:**

Open-ended interviews and Interpretative Phenomenological Analysis (IPA). The analysis involves both individual and more generalized investigations based on the contributions from seven participants.

**Findings:**

The findings show that music is an integrated and habitual aspect of the adolescents’ daily lives, used as a tool for emotion regulation, cognitive enhancement, and creating personal space where one can be free from outside criticisms and distractions. There is a preference for music listening in headphones which creates a more intense and private experience. There are varying levels of awareness of the potential hearing-health risks, but the profound meaning of music for their well-being often overshadows any concerns.

**Conclusions:**

Despite awareness of potential hearing-health risks, the adolescents prioritize the immediate emotional and cognitive benefits of music. Technological advancements and increased social media interactions contribute to a trend towards more personalized music listening. These insights call for more complex intervention strategies and models for health promotion which account for the positive aspects of music listening, instead of merely focusing on the potential risks of loud music.

## 1. Introduction

### 1.1. The meaning of music in adolescents’ lives

Music can significantly affect adolescents’ emotional well-being, social interactions, and identity formation. Research has shown that young people use music to fulfil specific roles such as mood and emotion regulation, stress management, enhancing social functioning, and creating meaningful memories (Beckmann, 2013; Groarke & Hogan, 2018; Miranda, 2013; Schäfer et al., 2013; Upadhyay et al., 2017). Further insights into the nature of these listening habits, particularly in relation to mood and emotion regulation, reveal that young people are highly aware of the impact their music preferences have on their mood. Many participants reported using music strategically; for instance, they often used classical music to induce calmness when feeling anxious, while upbeat pop music was used to uplift their mood during periods of sadness (Stewart et al., 2019). Music often feels meaningful to many people, even if that meaning is entirely personal (Cross & Tolbert, 2016). Beckmann (2013) showed that music can have a profound impact on how adolescents perceive and feel about the world, themselves, and their interpersonal relationships. Music is more than a form of entertainment or a passive activity, it is also used to cope with the highs and lows of life. Given its complex role in adolescents’ personal lives, social lives, and in creating a sense of meaning, Beckmann (2013) found that music should be recognized as a valuable source for health-promotion. Furthermore, music can play a pivotal role in the social lives and identity formation of adolescents. According to Miranda (2013), music provides a social medium through which young people connect, share experiences, and express themselves, often helping to forge and reinforce group identities and peer relationships. Music is not only a form of entertainment but also deeply intertwined with cultural and social contexts. Cross and Tolbert (2016) discuss how music listening is embedded within the cultural practices and social interactions of young people, reflecting their values and societal norms. While high volumes can have detrimental effects, moderate, and controlled music listening has been shown to have cognitive benefits. Schäfer et al. (2013) highlight that certain types of music can enhance concentration and learning efficiency in adolescents, suggesting that music has a place in educational settings. Music can also empower adolescents by providing a sense of control and independence. Upadhyay et al. (2017) illustrate how personal music choices allow young listeners to shape their environment, making it conducive to personal growth and self-expression.

Adolescence also constitutes a time during which young people are often concerned with how their peers perceive them (Vogel et al., 2010). Research indicates that young people develop their identity by experimenting with various behaviours and activities (Jiang et al., 2016). Identity encompasses both individual and social aspects, reflecting one’s connection to a collective or cultural identity, as well as a sense of self and personal growth. Culture, art, and music can provide powerful emotional experiences that shape and define identity (Beckmann, 2013). To identify oneself with a particular style of music in youth can lead to the formation of personal and/or group identity in which all members can unite in the choice of music and thereby establish and maintain relationships (Upadhyay et al., 2017). When seeking to understand the meaning of music within social contexts, it has been emphasized that music is not solely enjoyed for its aesthetic but is deeply intertwined with the social, cultural, and contextual aspects of its existence (Cross & Tolbert, 2016). Furthermore, adolescents are in a critical phase of neurodevelopment where their sensitivity to rewards may be heightened (Steinberg, 2008). This developmental phase can make loud music particularly appealing due to its intense sensory experience, potentially explaining why some young individuals prefer higher volumes despite being aware of the risks. However, as the brain develops improved cognitive control and emotional regulation skills, teenagers become better at managing and resisting impulsive and risky behaviours as they transition into adulthood. Therefore, adapting to health-promoting listening habits during this developmental stage could be a crucial determinant of young people’s future health (Jiang et al., 2016).

### 1.2. Music listening habits and hearing health

While music brings numerous benefits, there is an increasing concern about the hazards of recreational noise exposure, particularly among children and adolescents, through activities such as attending concerts, festivals, clubs, and listening to music through headphones—a practice that has grown with technological advancements in accessibility of social media and music listening platforms (Feder et al., 2021; Le Clercq et al., 2018; Paping et al., 2022; Wang et al., 2020). Questionnaire-based findings suggest that young people’s attitudes towards loud music vary depending on personal factors such as personality and perceived symptoms of NIHL (Gilles et al., 2013; Manchaiah et al., 2017; S. Widén et al., 2013). Adolescents listen to music with varying habits and preferences, highly influenced by their emotional and psychological states. Dillard et al. (2022) estimate that 23.81% of young people listen to music daily at volumes or durations above safe thresholds. Saarikallio et al. (2020) show that adolescents reported mainly listening to music in solitary settings, which allows for personal control over the listening volume and duration. According to Paping et al. (2021), the average adolescent listens to music approximately two days per week, with each session lasting about 81 minutes. The main devices used were smartphones and the majority of the adolescents favoured earphones (in-ear) over headphones. Volume levels during these sessions were generally moderate, with the mean listening level reported at about 54.5% of the maximum volume capability of the device (Paping et al., 2021).

High volumes and prolonged listening can cause symptoms such as tinnitus, temporary hearing loss, and sound sensitivity, potentially leading to permanent auditory damage (You et al., 2020). Despite adolescents’ awareness of these risks, the adoption of hearing protection remains low, with significant variations across countries. For example, only 14% of US adolescents use hearing protection at loud music venues, and only 1.6% usage reported in a Brazilian study (Chung et al., 2005; Zocoli et al., 2009). Furthermore, a study comparing Swedish and US adolescents suggests a country-dependent trend: Swedish youth were 12.8 times more likely to use hearing protection at concerts, with 179 participants from Sweden compared to 203 from the US (S. E. Widén et al., 2006). Beyond hearing health, exposure to loud music over an extended period of time can also affect quality of life, causing difficulties with communication, academic performance, as well as lower self-confidence among adolescents (Herrera et al., 2016; Lee & Jeong, 2021; Warner-Czyz & Cain, 2016).

In summary, while the risks associated with prolonged, and loud music listening can be significant, it is also important to consider the benefits and meaning that music can bring. As technology has made music more accessible, its impact on people continues to grow in significance, as such, music leaves much more to explore in the context of health-promotion (Beckmann, 2013). The positive aspects highlight the importance of engaging with adolescents to explore their personal experience and perceptions of the uses and meaning of music. By understanding the role that music plays in adolescents’ lives, strategies can be developed to encourage safe listening habits at an early age without diminishing the benefits that music provides, ultimately fostering health-oriented music listening behaviours (Gopal et al., 2019; Lee & Jeong, 2021). This approach fosters a deeper understanding of why adolescents might engage in risky music listening habits and how these can be mitigated through informed health-promotion strategies that resonate with their experiences and values.

### 1.3. Aim

The aim of this study is to investigate the role and meaning of music in the lives of adolescents and how adolescents understand the potential impact of music listening on hearing-health.

## 2. Materials and methods

### 2.1. Study design

This study adopts a qualitative approach as the primary goal is to develop a deeper understanding for why young people listen to music based on their own experiences. Subsequently, interviews were considered the most suitable method for data collection. Interpretative Phenomenological Analysis (IPA) is the method used to analyse the interview material. IPA is an analysis method which aligns with phenomenological philosophy (Smith et al., 2022). Phenomenology is a philosophical and methodological approach which prioritizes the study of subjective experiences and the meaning individuals attribute to those experiences. The aim is to capture the world as it appears to the participants themselves. IPA studies acknowledge the inherently interpretative nature of human sense-making and adopt an idiographic approach, emphasizing the in-depth examination of individual cases, rather than general ideas or theories. This method is also particularly well-suited for investigating how a relatively homogeneous group of individuals make sense of a shared situation, such as motivations for music listening. IPA was selected over other qualitative methods because of its emphasis on individual lived experiences and depth of exploration into subjective meanings of personal experiences. By studying individual experiences, IPA enabled a deeper understanding of the complex and personal incentives behind music listening among adolescents. Therefore, we used this approach to investigate why and how adolescents use music and what it means to them in their daily lives, providing detailed insights into their experiences and perspectives.

### 2.2. Sampling and recruitment

Letters containing information about this study were distributed in four different educational settings in Örebro, Sweden; one primary school, two upper secondary schools, and one cultural school (“Örebro Kulturskola” - organization that offers music and arts education to supplement regular school). One participant was recruited from the primary school and had an interest in pursuing a formal music education. Three participants were recruited from the two upper secondary schools; two of the participants majored in natural sciences, one majored in social sciences, and one majored in technology/engineering. Lastly, two participants were recruited from the culture school where they were enrolled in learning an instrument and were also majoring in arts/music in regular upper secondary school. Seven people were selected to participate in this study through purposive sampling. The findings contain contributions from all of them. Participants ranged in age from 15–19 years (4 females, 3 males). For participation in the study, individuals were required to be between the ages of 15 and 19, and to be capable of providing information relevant to the aim of the study. Consequently, the participants were expected to have daily or regular experiences of music listening. Exclusion criteria encompassed individuals with diagnosed hearing loss. Individuals with diagnosed hearing loss may use hearing aids and/or may not engage with music in the same manner as those with typical hearing function. The age range of 15–19 years was chosen due to adolescence being an important period in which music listening habits are shaped and established, this may in turn have an effect on hearing-health and well-being later on. Furthermore, older teenagers have likely been listening to music for a few years longer than younger ones, which is relevant to their level of experience and their ability to express the significance of music. We aimed to achieve a mix of genders. While this study does not focus on generalizing findings based on gender, it is essential to incorporate a variety of perspectives. Given that few participants are included in phenomenological studies in general, it becomes important to include individuals with unique backgrounds, including variations in age and gender.

### 2.3. Ethical considerations

The study was not subjected to formal review, but the Swedish Ethical Review Authority raised no objections to the study (Ref: 2021 -05,694-01). Participation in the study is based on voluntary participation and informed consent from the research participants. Those interested in participating read the information letter in their own time and then contacted us. We then asked questions relating to the eligibility criteria and scheduled an interview. Upon meeting, we revisited the information letter and asked if they had any questions, they were then asked to sign a consent form. Research data is handled in accordance with the General Data Protection Regulation (GDPR), interview material and transcriptions are securely stored, pseudonymized, and only accessible to relevant project personnel. The results will be presented in a way that prevents the identification of individual research participants.

### 2.4. Interview process

Seven open-ended interviews were conducted, tape-recorded, transcribed verbatim and the transcribed material formed the basis for the analysis. All meaningful spoken content, including words, pauses, non-verbal expressions, and emphasis on specific words, was noted during the transcription process. In an IPA analysis, data collection methods that create conditions for the individual participant to provide as rich and detailed a description of their experiences as possible are suitable, hence the open-ended interviews employed in this study (Pietkiewicz & Smith, 2014). The interviews were conducted by the first author of the study to maintain consistency across all interviews. The interviewer had no prior acquaintance with any of the participants as the recruitment process involved informing potential participants about the study through announcements made in large group settings, such as classes or student gatherings. The interview process was designed to explore the depth and variety of each participants’ engagement with music. To achieve this, an interview guide was constructed with the ambition to formulate areas of questioning that were as open-ended as possible. More specific so-called “trigger questions” or “prompts,” were also prepared in case any participants had difficulty providing rich descriptions of their experiences (Pietkiewicz & Smith, 2014). Ten question areas were the basis for the interview guide, some of them included one or more prompt. All the question areas had one main question which was posed to all participants, whereas prompts and follow-up questions were different depending on the conversation. For example, one question area was “The meaning of music,” where the question was “Talk about what music means to you,” and the prompt was “What thoughts and feelings come up when you think about the meaning of music in your life?” For the interview guide in its entirety see appendix B. The interviews lasted forty to seventy minutes per participant. The questions were asked in different orders depending on what was considered appropriate for the flow of conversation. All question areas were covered with all participants.

## 3. Analysis process

**Figure 1.**
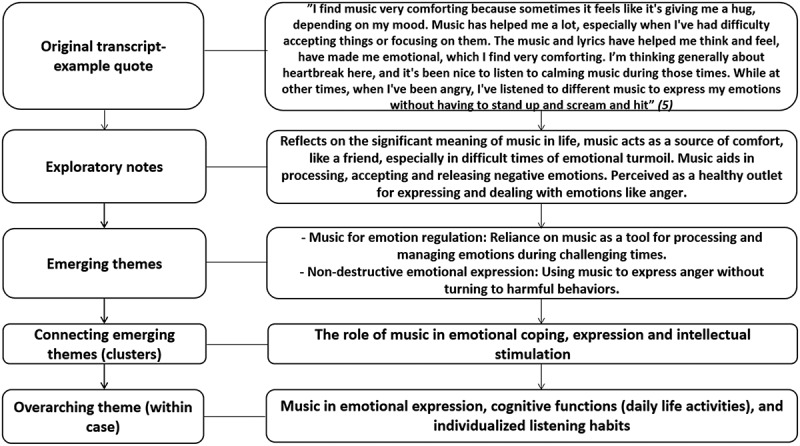
Example of the analysis process within a case (steps 1–5). Showing the process of analysing one case which was a part of creating the resulting (group level) superordinate themes.

**Figure A1.**
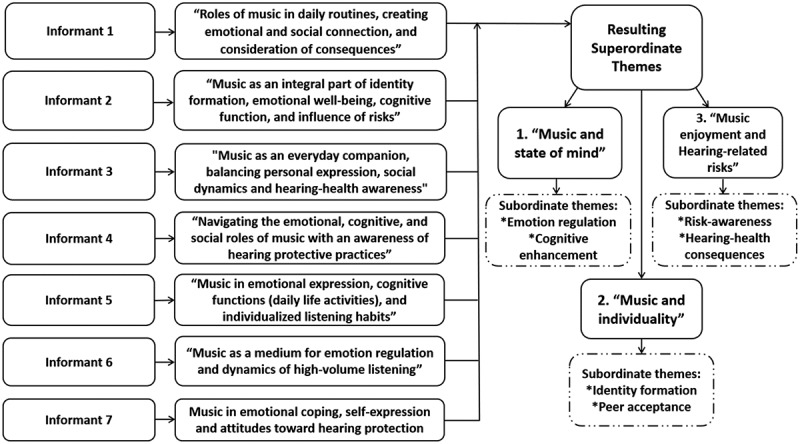
Example of the analysis process—across all participants (step 6). Individual overarching themes that led to creating the resulting group level superordinate themes.

All data analysis was performed by the first author manually, following the steps described by Smith et al. (2009). For examples of the analysis process with excerpts from the transcripts, see Figure 1 below and Appendix A (Figure A1). Before listening to the recordings, the first author engaged in bracketing; noting and setting aside preconceptions and assumptions.

After the interviews, the first author listened to the recordings and noted initial thoughts simultaneously. Thereafter, the transcribed material of each interview was closely examined, and further exploratory notes were made. The phenomenological aspect in this step focuses on generating an understanding of how the participants express the explicit meaning of their experiences. Noteworthy linguistic expressions such as emotionally charged phrases were also noted, emphasizing indications to explore underlying emotions, motivations, and meanings associated with music.

The initial coding process involved breaking down the transcribed data into smaller segments, with each segment assigned a short phrase (code) that captured its essence. These were then reviewed and grouped based on conceptual similarities to develop broader themes. Each theme represented a significant aspect of the participants’ experiences.

Emerging themes were examined for patterns and connections. These themes were grouped into clusters based on conceptual similarities, with the emerging themes moved to a separate document for an in-depth review. This process eventually led to larger overarching themes in each individual case. This approach helped in understanding the overarching patterns within and across participants’ accounts.

The overarching themes from each individual analysis were compiled into a common document. These themes were studied to identify central group patterns aiming to capture all lived experiences, moving back and forth between in-depth individual details and the broader context. The final group-level superordinate themes are presented in the findings section and in Figure A1 (Appendix A). The analysis process guides the selection of demonstrative quotes and analytic interpretations presented in the findings. The final step involved using the themes to create a cohesive narrative, supported by verbatim quotes from the participants.

Themes were identified based on their prevalence and significance within the data, including how often a theme appeared across different interviews and the depth and detail provided in the descriptions.

To ensure the trustworthiness of the analysis process, several strategies were implemented. Themes were cross-checked and validated by the co-authors who reviewed the transcripts and the emerging analytic content independently. Detailed documentation of the analysis process was kept providing a trail of how themes were reached. The full interview guide, included in Appendix B, provides a set of questions designed to prompt detailed descriptions of the participants’ experiences with music.

## 4. Findings

The findings will be presented under each superordinate and subordinate theme as shown in Table I and each participant will be represented by a number (e.g., P1-P7). The music listening habits, based on the participants’ statements, illustrate patterns of both individual and shared experiences. Music is described as a near-constant companion for these individuals. Whether commuting to school, engaging in physical activities, or simply transitioning between daily tasks, music is more or less present. The participants found it challenging to precisely quantify the number of minutes or hours per day or week they spent listening to music. However, from their detailed descriptions, it can be reasonably inferred that their engagement with music is highly frequent. For instance, all participants noted the presence of music during bus or bike rides to and from school and workouts, during the workouts, while studying, and one participant reported sometimes listening to music while sleeping as well. This suggests that music is integrated with many or all of their daily activities. One vivid example involves a participant who uses music as part of their morning routine, as well as during showers, although they prefer instrumental songs in the shower to suit the acoustics and their ability to hear. Another one of the more prominent disclosures comes from a participant who estimated roughly how many hours per day they listen to music, as explained in the following quote:

**Table I.** Findings: superordinate themes with subordinate themes.

Superordinate themes	Music and state of mind	Music and individuality	Music enjoyment and hearing-related risks
Subordinate themes	*- Emotion regulation* *- Cognitive enhancement*	*- Identity formation* *- Peer acceptance*	*- Risk awareness* *- Hearing-health consequences*

I listen to music every day. For example, I wear headphones when I bike to and from school. I listen when I am at home. Last year, I think I listened for about 85,000 minutes, according to what you can see on Spotify, so that’s quite a lot, several hours per day. (P5)

Three of the participants were enrolled in music education, such as learning to play an instrument, playing instruments in orchestras/bands, or singing in choirs. Their weekly routines include participation in orchestra, band, or choir practice, playing instruments after school/music education, as well as more passive music listening after school. The preference for listening to music in headphones emerges strongly among the group. The adolescents describe striving for a balance between a comfortable volume setting and still being able to appreciate all elements and instruments in the music. Additionally, music often serves as a study aid for participants within the group, though preferences vary widely. For instance, instrumental music is favoured by some for its ability to help the individual maintain focus during tasks, while others find that music with lyrics can be too distracting for activities like reading or writing. Nevertheless, some form of music, or occasionally other audio content, such as podcasts, is commonly present during study sessions both at home and at school on a daily basis.

Key elements within and between all participants’ narratives indicate that music is not only an everyday activity and constant habitual companion, it also fulfils specific purposes. It functions as a form of escapism or way to fill tedious and quiet moments in daily life. Music also helps with emotion regulation and reducing overstimulation and overthinking. All participants show a preference for listening to music in headphones as the music sounds more vibrant and it offers a more immersive and personal listening experience. Another main use of music and headphones for all participants is to create a “bubble” of personal space within their public surroundings, allowing them to avoid social interactions or disturbing environmental noise. While there is a general awareness of some potential risks to hearing-health due to music listening, it seems that the profound meaning of music in their lives often takes precedence over any concerns.

### 4.1. Music and state of mind

The findings from the analysis show that the informants rather expertly use music for self-care in everyday life. Music functions to regulate emotions and moods, offering a way to be alone in various contexts as it creates a private space for the individual to focus on their emotional well-being. Music acts as a multifaceted coping mechanism to manage, feel, and express emotions, to change or enhance certain moods, and for stress reduction. Thus, this theme reveals the therapeutic role of music in the participants’ lives as they display a profound personal connection with music which often reflects their emotional state. Individuals may for instance select songs or genres that match their mood, allowing them to process their emotions and depending on the music and their goals, it can also have either a calming or energizing effect. Furthermore, music can have an impact on various cognitive processes; it can increase or decrease concentration, focus, and motivation, enhance productivity and happiness during daily activities, and help individuals mentally escape from reality during stressful situations.

#### 4.1.1. Emotion regulation

The adolescents emphasize that music provides comfort in challenging times, elevates their mood, and contributes positively to the overall experience of daily life. The adolescents imply an awareness of the impact of music on their emotions and hence a strategic use of it to regulate their emotions, such as listening to soothing music to reduce anxiety, or upbeat music for a mood boost, or as one of the adolescents expressed it:

I become much happier and in a better mood quickly if I listen to music. You relate, you go along with it, you listen to a song you like, or an artist you like, and it makes the situation better. It’s much more enjoyable to do something while listening to music at the same time. It puts you in a better mood. (P1)

The adolescents intentionally use music to distract from negative or overwhelming feelings and thoughts. There are particular mentions of using music to cope with stress, ultimately allowing a shift of focus from a more negative state of mind to a more positive. The participants select specific songs to match their emotional needs and this process is deeply personal and tailored to their unique music preferences. This is an indication of the connection between music and productivity during stress or emotional distress as the music allows them to cope with difficult emotions while simultaneously maintaining the functionality required to engage in certain tasks, as exemplified by the quote below:

If you feel something specific, you can listen to music that enhances that feeling. It often happens when I’m stressed. I tend to feel down and don’t want to handle anything. In those moments, you can think that you’re allowing yourself to feel that way, but then you can move on and get something done. You don’t dwell on it for too long or prolong it. It’s also about making a change. I have a lot of music just to improve my mood. Sometimes there are those feelings you can’t quite describe, and you just want to forget about them for a while. Music makes it very easy to think differently. (P2)

Furthermore, music can create a sense of understanding as it helps the adolescents to deal with emotions that they might struggle to identify, cope with, and articulate. As such, music is utilized as healthy way to process and regulate emotions like anger, in which case, calming music serves as a form of self-control. When personally resonating to certain songs, it can show people that they are not alone, and that other people experience similar difficult problems, leading to a sense of comfort, belonging, and understanding, as indicated by the following quote:

… I find music very comforting because sometimes it feels like it’s giving me a hug, depending on my mood. Music has helped me a lot, especially when I’ve had difficulty accepting things or focusing on them. The music and lyrics have helped me think and feel, have made me emotional, which I find very comforting. I’m thinking generally about heartbreak here, and it’s been nice to listen to calming music during those times. While at other times, when I’ve been angry, I’ve listened to different music to express my emotions without having to stand up and scream and hit …. (P5)

Overall, this subtheme illustrates that in moments of emotional and inner turmoil, music becomes a companion that helps the adolescents to process, reflect, and experience their emotions more deeply. It also becomes a source of solace, especially during heartbreak. Conversely, when feeling anger or frustration, music provides a way to express it without resorting to physical reactions. This is an example of the role of music as a therapeutic tool, illustrating its capacity to facilitate emotional expression, self-regulation, and connection with others who share similar experiences. In summary, music is a unique and valuable resource for the adolescents to use when navigating complex emotions during challenging life events.

#### 4.1.2. Cognitive enhancement

The influence of music on concentration is evident in the adolescents’ experiences, they express that music can either improve or worsen concentration and focus depending on the setting and goals of the activity. Music can not only enhance productivity and efficiency, but also increase happiness during routine activities and tasks, essentially making them easier to get through. There is however a preference for studying in silence for some of the participants, as the silence enhances their ability to focus on the work, and in that case, music can be detrimental to concentration, as seen in the following quote:

When I’m studying, I can find it nice to listen to music or podcasts, but it can also be a bit irritating and distracting. If I need to focus on something else, having music can be distracting at times. I think I focus better in silence. I can’t handle any song when I’m studying; it needs to be something else, like someone talking or a podcast. But I’m not sure if I can really deal with music; it takes away my focus when I’m doing something else. (P1)

Music can also act as a protection from external distractions, such as in a loud classroom setting where one’s focus can easily be interrupted. Whether the goal is to feel more calm or more focused, music serves as a versatile tool that can contribute to creating an internal feeling of peace in stressful situations, as exemplified by the following quote:

It’s that I can distance myself from other sounds. It [music] can help calm situations and keep me calm, and it helps me try to relax in stressful situations. Instead of having all these sounds all the time, you stay in your own little bubble. It is a tool to be able to focus. Otherwise, with the sounds again, like in a classroom, I hear all of them, and I focus on them, and then my concentration goes right out the window. (P7)

Music can also make cognitive tasks easier when working on school subjects which can be perceived as more challenging than others and require intense thinking effort. The music makes these situations easier by providing a form of escapism, relief in mental effort and by eliminating some of the underlying thinking or anxiousness that may exists, which in turn makes it easier to focus and solve problems, as shown in the following quote:

I listen a lot when dealing with difficult subjects, like math and science. You have to think so much, and it gets exhausting. So, you can think that with music, it enhances the process. It’s as if it takes away some of that underlying bothersome thinking that’s usually there. (P2)

As much as there is a reliance on music as a catalyst for productivity, ultimately helping with accomplishing work and tasks more efficiently, there is also an emphasis on accomplishing this while simultaneously maintaining a good mood and clear mind. As exemplified by the quote below, it is not always about actively paying attention to the music, it is simply by having music there as a “background thing” that can be helpful in sustaining flow and organization of thought while multitasking:

I usually get ready and listen to music at the same time. I find that, perhaps not that time passes faster, but it feels much easier to get things done. Things become more automatic, and if I don’t need to focus, I can accomplish tasks without pausing or overthinking. I just choose my clothes, apply makeup, pack my bag, and everything flows smoothly because I have a “background thing” that holds everything together. So, I feel efficient, and it makes me happy. (P4)

In summary, the exploration of the influence of music on well-being across the seven participants’ experiences has revealed an intricate relationship between music and the individuals’ state of mind, stressing its therapeutic value and powerful role in cognitive enhancement, suggesting that the adolescents’ existence is more enjoyable and easier with music in their lives.

### 4.2. Music and individuality

Music can be used for retreating into an inner world, but it can also be used to create social connection. However, an individualized engagement with music and the private space it can offer seems to be more important for the adolescents, even if it makes communication and connection with others more difficult. Music is an integral part of how the participants define themselves as well as relate to others through their musical preferences. Their experiences often reflect a vulnerability in sharing music preferences with others, and a fear of judgement from their peers. Whether it is a positive or negative experience to share one’s music preferences with others, there is an emphasis on the profound role that music plays in life as it is a source of happiness that has become a fundamental part of their existence.

#### 4.2.1. Identity formation

Musical identity can include personal preferences for specific genres, artists, or styles. Music can be a conduit for the participants to express themselves and their interests. It can have a powerful impact on the adolescents and their sense of self, as one participant expressed it, “I feel happy when I listen to music. It means a lot to me because it feels like it’s a part of me now, as I listen so often” (P6). This indicate that music has had a role in shaping who they are. The adolescents also show a journey of musical exploration and self-discovery through musical interests. While their music preferences may change and evolve over time, the passion for and personal connection to music remains a constant thread. Their experiences also indicate a reluctance to share music with others as musical preferences can be deeply personal and sometimes feel embarrassing to share, especially as a young teenager, as seen in the following quote:

I went through different music phases when I was younger. In my first music phase, I listened a lot to video game music and instrumental music because I thought it was cool, but I found it really embarrassing when my mom and dad asked me what I was listening to, so I never talked about it. But then I started listening to a lot of rap, especially Eminem. Then, from around ninth grade, I began listening to rock and metal …. (P5)

Music is understood as a reflection of identity and personality and this perspective illustrates the importance of staying true to who they are and following their passion when making decisions about their lives and shaping the future. The following quote shows how a participant feels that no other academic or career paths resonates as deeply as music:

I thought I would feel at home in the arts program. I considered going for science, but I don’t know, there’s nothing else that I felt would be like “this is me as a person”, I wouldn’t say “I would love to discuss biology or chemistry with my friends,” instead, I preferred to talk about music and instruments. (P4)

Discourse surrounding music can be significant in friendships and social interactions. This highlights the deep emotional connection with music preferences and the vulnerability experienced when someone criticizes one’s personal musical preferences. The strong emotional investment in music suggests that music plays a significant role in the sense of self, which means that criticism may feel like a personal and emotionally charged attack, as one of the participants explained, “Because music is a part of me, so if someone criticizes the music I like, it feels like a personal attack. That can make me upset” (P7). These experiences collectively exemplify that the participants choose music that resonates with their emotions, experiences, and values. When someone criticizes their musical preferences, it can feel like an attack on their identity, leading to a sense of vulnerability and making them act defensive. The connection between music preferences, friendships, and social interactions can be complicated. It involves elements of personal identity, emotional attachment to the music, as well as shared experiences and memories. Criticizing someone’s music preferences can have a profound impact on their emotions and the dynamics of their relationships.

#### 4.2.2. Peer acceptance

This subtheme explores the pressure to conform to certain norms and expectations while simultaneously desiring individuality. There is a sense of an urge to fit in and be accepted by peers, which can sometimes lead to a reluctance to openly share unique music preferences. This reluctance may stem from the fear of being judged or criticized by others for having music tastes that are perceived as unconventional or personal. The tension between being “different” and “fitting in perfectly” highlights the struggle to find a balance between conforming to expectations versus asserting individuality during adolescence. One of the participants explains finding solace in going to music class as it is one of the only places which provide a safe space where one can freely express and discuss unique music preferences and interests, as exemplified by the following quote:

I’m in high school, and of course, you’re supposed to be different, but you’re also supposed to fit in perfectly. But it’s nice to be in a music class because then at least you can talk about music as you want. You should know things about music, but you can still have your own opinions. (P4)

There is a recognition that in the pursuit of peer acceptance, there can be a tendency to choose more mainstream and safe music that aligns with what is considered socially acceptable. This choice can be a protective measure, allowing individuals to avoid potential negative reactions or exclusion from their peer group. Although individuals might be passionate about their favourite music, they are not always confident in sharing it openly. The following quote shows that there can be a sense of discomfort with certain genres due to a belief that some genres are more socially acceptable while others are more “embarrassing”:

For example, in Swedish class, we have “Friday songs,” and I always think about which song is the most neutral one I can show so that no one judges me. I sometimes have these situations where I get very uncomfortable about showing what I listen to, even though I love my taste in music. If I had been listening to local rap, I probably would have found it a bit embarrassing”. (P3)

Several experiences with music in social contexts are marked by this tension between personal preferences and peer preferences. The individual makes effort to navigate these differences while acknowledging the challenges and emotional responses that follow. However, despite the efforts to adapt to group preferences, there can be frustration and unhappiness due to the music preferences of others. This highlights the emotional impact of music-related differences between people. The response to this frustration seems to be to turn inward, keep their music preferences private, and only listen to it while alone, as shown by the quote below:

In the social contexts I encounter, very few of my friends listen to the same music I do, so I’ve always adapted. I just have to accept that we don’t listen to the music that I appreciate and enjoy. But it also bothers me at times that it’s only their music that I sometimes find boring and, in some cases, really bad. So, in social contexts, I’ve mostly kept my music to myself. (P6)

Another motive for keeping music preferences private can be that music often reflects one’s mental health and emotional state. While being open to finding new music to enjoy, there are some reservations about doing so publicly due to previous experiences of being judged or ridiculed by those who do not understand or enjoy the same music. The quote below illustrates that there is a desire to only share music with close friends, this could be a strategy to avoid judgement and sustain a sense of belonging within specific peer groups:

Because my music very clearly reflects my mental health, it’s very personal. And I’m very open; sometimes I listen to odd music. Many people are into what’s “in” right now, and right now, it’s a lot of hip-hop, for example. I can listen to hip-hop, and I like hip-hop. But a few months ago, for example, I was listening to Milky Chance, and there were some guys who said, “What the hell is that?” And I said, “It’s a German rock band,” and it became something to laugh at. And then I just feel like, no, I shouldn’t expose my music. I want to keep it as much to myself as possible for those who aren’t close; they have nothing to do with my music. (P7)

The adolescents often listen to music for the purpose of transcendence and being in their own world, they use headphones strategically for that same purpose, as well as for signalling to others that they want to be left alone. Music is often used as a form of escapism, particularly to disengage from the stressors of social gatherings and events, one of the participants explained it as being a source of solace and comfort in those situations, “I listen to music even more if I’m at a social event; I completely disconnect, and I prefer not to hear any voices.” (P7). Music can also serve as a temporary refuge to disconnect from reality in general and to have music as a backdrop to certain moods. Beyond that, the adolescents seem to hold an idealized view of music as a solitary activity, and there is a perceived uniqueness or specialness in having alone time with music, or as one participant expressed it:

It’s about wanting to disappear from the world, but also about feeling a bit romantic in your own little bubble and feeling special. I might not relate to the lyrics so much, but the music might fit very well with a certain day, for example, if it’s autumn and cozy, and the music suits that. (P4)

While trying to preserve solitude in music listening, using headphones seems to be the most common method to achieve it, especially in social settings or public places. When an individual visibly wears headphones, it is a sign to others that they should not attempt to approach the individual, and the adolescents understand this behaviour as a form of social etiquette. They hold a belief that personal boundaries should be respected in this kind of scenario, as seen in the following quote:

… It might be the case that I’m not listening to music but just have headphones on because I don’t want to talk to anyone or be with anyone; I want to be by myself and get away. That’s what I think, anyway. When I see someone with headphones and I don’t know how the person feels, it could just be that they’re listening to music for fun, but I tend to think that if I see someone with headphones, I usually don’t go up and talk to them unless it’s very important because in that case, they probably don’t want to be approached. (P1)

Headphones can also be used to endure overwhelming social situations by shutting out external sounds. This strategy suggests that the adolescents consider whether they should or should not wear headphones during real time social events or even at home, giving themselves a choice between socially engaging or retreating into themselves, as seen in the following quote:

… I’m somewhat sensitive to sound, especially to too much noise at the same time. So, when there are large groups of people moving from one place to another, and many people talking, I just want to put on my headphones and block out all other sounds. And often at home, I like to be in my own bubble, and then headphones help a lot because it’s easier to talk to someone who doesn’t have headphones on than to talk to someone who does have headphones. So, it signals that I’m in my own little bubble. (P5)

Music can influence social engagement in conflicting ways, it can contribute to a social situation in a way which makes connecting to people easier and more casual, but it can also be distracting at the same time. Due to the accessibility of it, there is a belief in the power of music as a social connector that holds universal relatability, allowing individuals to communicate and relate to each other easily. The quote below demonstrates how music can create a sense of unity and inclusivity:

Music can be like this; it can be distracting in a situation, taking your focus away from what you’re supposed to do, but it can also be great for bonding, you can bond well through music. It’s easy, if things get a little awkward, you can put on some music, find common artists and songs you both listen to and like. It’s very easy to make friends through music, whether when meeting new people to play music with or getting help, it’s a great community. You always have something to relate to with music. (P1)

Expanding further on the multidimensional influence of music, both the individual and collective experiences indicate that music can be associated with togetherness and have a positive influence on family bonding by contributing to create certain sought after atmospheres. However, it can also be seen as a barrier to connection and communication, especially in relation to headphone use, leading to a sense of exclusion and disconnect, as seen in the following quote:

I like to play music when I’m cooking with my family, for example. Yesterday, we listened to a Lucia playlist while setting up for Advent, and it was cozy; we all got a Christmas feeling. I think it helped; otherwise, we might have become more frustrated when we couldn’t assemble our angel chimes. I find that it helps a lot. But often at school when I talk to others, they have music in their headphones all the time, and I know they don’t get as distracted as I do because they can talk to me at the same time. But you feel a bit excluded, like they’re experiencing something more, and maybe not entirely focused on me. (P4)

To summarize, this subtheme shows that music plays a multifaceted role in adolescents’ personal and social lives, reflecting their navigation between staying authentic to themselves and their interests on one hand and peer acceptance on the other. It also shows that music can have opposing effects in similar situations, as it can be useful in creating a safe isolated space or facilitating social connection, but it can also hinder communication and connection.

### 4.3. Music enjoyment and hearing-related risks

This theme reflects the adolescents’ understanding of hearing-health risks in relation to music. Their experiences lead to exploring the complexities of loud music listening, where the enjoyment of loud music competes with the awareness of hearing-health risks. The participants exhibit varying degrees of understanding the associated risks, which in turn influences their music listening behaviour. Furthermore, there seems to be a limited understanding of the relationship between long-term consequences to hearing function and loud music exposure. While some of the participants seem to have at least an awareness of this connection, it does not necessarily translate into any concern or modification in their music listening behaviour.

#### 4.3.1. Risk awareness

This subtheme explores the adolescents’ perspectives on health risks associated with enjoying loud music or in relation to prolonged music listening. It appears that the adolescents derive greater enjoyment and satisfaction from music when it creates the intensity and energy that loud music can deliver. The collective experiences reflect a complex interplay between awareness, behaviour, and emotional connection when it comes to enjoying music. While the participants may be aware of the potential risks, it can be challenging to consistently keep the volume level at a lower intensity, or to be concerned about hearing-health risks which are often described as difficult to grasp. The main motivation for enjoying loud music lies in the sensory and physical experience of it, it creates an overall positive response in the individual and their mood. Loud music often makes the music experience better, and creates a more immersive feeling both physically and emotionally, as one participant explained it:

I love having loud music because it feels like if it’s not at high volume, it’s almost not the same thing. It sounds better and feels better when it’s loud. It’s about the music experience, and it feels good in the body. (P6)

Beyond the perspective that high volume makes music sound and feel better, it can also make normal daily activities feel more enjoyable. The adolescents seem to consciously regulate the volume depending on specific contexts and situations. The following quote shows an understanding of the impact that music and high volume can have on their mood, motivation, and concentration, thereby using it to achieve different goals:

If I’m coding or gaming, I primarily use the speaker, and it’s just nice to feel the bass or vibrations from the music, which leads to me increasing the volume. Meanwhile, with headphones, the volume is mainly to block out external sounds. But it definitely creates a different experience, and it also depends on whether I’m aiming for just the music experience or focusing on reading. In that case, I might lower the volume quite a bit or even turn off the music at times. (P5)

Increasing the volume can make the music feel like a new entity, which makes loud volume a key factor in altering musical experiences. The experience that high volume makes music feel “completely new” indicates an excitement and feeling of novelty in experiencing familiar songs in a different way. The consistently similar descriptions from all the participants of music as “fresher”, “crisper” and “more real” due to loud volume emphasizes the importance of heightened sensory engagement, which may also amplify the emotional impact, as described by one of the participants, “It’s like the music becomes entirely new, as if you’ve dusted it off. It feels fresher and crisper, more real, as if I had a live band in my ears” (P4). Hearing-health risks are seldom at the forefront of the adolescents’ minds, it is apparent that the appeal of music and the immediate pleasure it brings is often prioritized. The passion for music and the enjoyment derived is one of the most central factors both on an individual and group level, as indicated by this quote:

It’s that you’re quite immersed in the music at that moment when you listen or play. I don’t think the first thing that comes to mind is hearing; at least, I don’t. I mostly think about the music, how it sounds, how it’s structured, what the lyrics are, you enjoy it more at that moment. (P1)

There is some awareness about the potential risks, but limited knowledge about the consequences to hearing-health associated with high-volume listening. There are however some experiences of attempting to balance the enjoyment of loud music versus protecting one’s hearing. The following quote is one of several that shows an example of a personal justification for the choice of listening to music at a high-volume while being aware of the risks:

I need to have a very high volume because otherwise, I can’t hear properly at all, and then I don’t shut out the other sounds in the same way. So right now, I mostly listen at maximum volume. I don’t know if it’s really good for my ears, but it’s to shut out all the other noises. If I have headphones on, I still have it loud. I always have a high volume. (P7)

There is variability in how the adolescents think about loudness and in their tolerance for loud music. Their experiences highlight the potential for individuals to unconsciously downplay the risks associated with loud music when an increased tolerance for loud sound levels is not understood as a negative outcome. For example, there is a belief that repeated exposure to loud music can help “train the ears” to handle higher volumes better. This is an indication of limited knowledge regarding the long-term consequences to hearing-health due since the notion of “tolerance building” seems to have a positive connotation rather than negative. As seen in the example below, the participant believes they can tolerate loud music for a longer period of time or at a higher volume before any discomfort is experienced, compared to others who may find it uncomfortable sooner:

Everyone is different; you can play music every day and still consider it to be loud volume without it being so loud. Everyone has different opinions on what is loud and bad. But I believe one becomes more accustomed when playing music every day, not just listening to music with headphones but playing and listening to real live music every day. Then it becomes easy to practice and develop tolerance for loud music. I think I have a higher tolerance before I feel like loud music becomes uncomfortable. (P1)

In summary, this subtheme explores how the adolescents’ make sense of the appeal of loud music and its impact on their emotional states. While they are somewhat aware of the hearing-health risks, the instant and powerful enjoyment of loud music is more important in the moment, and there is a seemingly limited understanding of the potential long-term health consequences.

#### 4.3.2. Hearing-health consequences

This theme contains perspectives on the short and long-term consequences of taking risks in music listening. Some of the adolescents reveal some awareness of the potential risks of listening to loud music regularly, but they mainly express a need for receiving more specific information about those risks and the real effects of cumulative noise exposure. There is some uncertainty expressed about the effects that could potentially arise from “just listening to music,” as illustrated by the following quote:

Of course, it’s not good to listen to loud music every day. If you do it over an extended period, I feel that it might increase the risk of some sort of hearing damage, but I don’t know anything specific that would come from just listening to music. (P4)

Additionally, there are some experiences that indicate their beliefs about what could happen to their hearing-health and the short term versus long-term consequences of exposure to loud music, both in the context of concerts and daily activities. The following quote shows an awareness of the possible short-term effects such as headaches and ear-pain as well as for the potential long-term effects, such as tinnitus and hearing loss:

In the short term, if it’s been too loud at a concert, it can be uncomfortable; you can get a headache and ear pain. If it continues for a while, in the long term, if you’re constantly surrounded by high volume and music every day and don’t think about it being too loud, don’t put on ear protection, you can get tinnitus and reduced hearing …. (P1)

There is an expressed difficulty in understanding how a minor increase in volume can impact hearing-health in a tangible way over an extended period. This is an example of the fact that the adolescents do not actually know how much of an increase in decibels that each volume increase on their smartphones corresponds to. Furthermore, perceiving minor increases in volume as insignificant may reflect a tendency to downplay the risks when there are no immediate or noticeable effects in hearing-health, which makes it more difficult to grasp that daily habits could lead to negative long-term consequences, as exemplified by this quote:

It’s difficult when it comes to the long term; it’s hard to grasp this tiny little increase in volume and how it affects you in the long run. I think it’s very difficult to truly comprehend. If I were to think, “okay, I have decent hearing at the moment, and I want it to last for this duration,” and then I would receive a set of guidelines on how to protect my hearing. But it’s easy to exceed those guidelines and think it’s not a big deal, even though it has long-term consequences. It’s hard to grasp it, even though I know the risks are there and what it might eventually lead to. (P5)

The participants sometimes show a higher level of awareness and knowledge of the potential hearing-health risks associated with their music listening habits. Even so, there is an emphasis on the importance of sensory gratification and emotional connection to music. However, based on that awareness, there is an attempt to find a balance between enjoying music and preserving hearing-health, as illustrated by the following quote:

I’m very aware that I don’t want to damage my hearing because it’s something I often think about when I play the drums. I always wear earplugs because the sound is incredibly loud. On my phone, there’s a feature that, when you raise the volume above a certain limit, marks it with a red symbol to indicate its very high volume. It’s something I strive never to exceed. But when it comes to metal music, there’s a sensation with the loud music; you feel it throughout your whole body. It’s a very delightful feeling, but in the long run, I think it can be quite dangerous for hearing to expose yourself to high volume for extended periods. (P6)

There is a basic and seemingly intuitive understanding that it can be harmful to listen to loud music. Nevertheless, there is also an admission of not being well-informed about why loud music can be harmful and an expressed need for tangible examples of the potential consequences that would make sense to them in their lives. This indicates that without deeper understanding, the adolescents may dismiss warnings about loud music as irrelevant, as seen in the following quote, simple advice is not necessarily enough to encourage a change in behaviour:

I’ve learned the message itself that you shouldn’t listen to music too loudly. It might have been more helpful to know why and what it leads to, so you understand how it harms you and really avoid it. Otherwise, you end up thinking: ‘they said I shouldn’t do it, but it doesn’t matter, it’s not a big deal. (P4)

The fear of developing hearing loss slowly over time leads to a strong emotional response and accentuates the cumulative nature of noise-induced hearing loss. Some of the participants show a conscious effort to integrate this concern into their lives, leading to a sense of caution. Their love for music and desire to preserve the ability to enjoy it signifies the meaning of music in life. The following quote reveals a deep sense of vulnerability and fear related to the gradual loss of hearing which is based on a strong will to continue enjoying music through life:

I haven’t felt any ringing in my ears, but after the concert, my ears felt tired, and it was like all sounds were a bit muted, but it went away overnight. It’s not something that has worried me … . I haven’t been concerned about that feeling. But sometimes, if I, for example, hit a cymbal and it hurts my ear, I think I shouldn’t do that again. However, something I’m a little afraid of is gradually becoming deaf without noticing, so I won’t know how my hearing has been affected because I haven’t personally noticed it, and it has just happened. It would be terribly sad if it turned out that way. I’m somewhat aware of it in my daily life; I try to think about it in the long term. I listen to a lot of music and want to keep listening to music; I don’t want to lose that. (P5)

Even among the adolescents who show awareness and some knowledge of the risks, there is a tendency to place less weight on future consequences compared to the immediate rewards of music enjoyment. This is reflected in the following quote, where despite risk-awareness, they frequently listen to music on maximum volume, which suggests a relaxed attitude towards potential future hearing-health consequences:

It wears down your hearing, I know that for sure. You’re supposed to keep it below 80% with noise-canceling headphones. I don’t. I keep noise-canceling at its maximum. But I’m aware it’s harmful, yet I choose to ignore the risk because… well, I don’t really know why I just disregard it. I don’t focus too much on “looking ahead,” like planning for the future. Instead, I try to live in the moment; I don’t have the energy to worry. I think if my hearing goes, it goes … I try not to worry about things too far in the future because, in reality, none of us knows how long we have, so why live with constant worry. (P7)

When reflecting on their levels of awareness regarding loud music exposure, the risks are seldom at the forefront of their minds. They do not typically think about their hearing-health, whether listening to music or not. Overall, the risks only become important in situations where the volume becomes painfully or uncomfortably loud. This reflects a situational and reactive pattern of behaviour, where negative physical consequences of loud music (such as tinnitus or ear pain) act as triggers to consider their hearing-health, or as one participant expressed it:

No, I don’t think about it much. If it ever gets too loud, I just think in the moment, “Now it’s too loud.” Otherwise, I don’t really think about my hearing very often; it’s more like if it gets too loud, you might think, ‘Oops, that hurt. (P1)

Knowing someone who suffered hearing-health damage due to loud music can also act as a trigger to be more cautious and mindful. The fear of experiencing similar problems becomes a catalyst for adopting more health-oriented music listening habits, for example by using the recommended volume limit that smartphones suggest automatically. However, there is a willingness to renege on this cautious behaviour and still increase the volume for certain songs, for instance their favourite songs. This once again shows a desire to find a balance between preventing hearing problems and enjoying music to the fullest, as reflected in the following quote:

Now, I’m somewhat fearful of playing my music too loudly because I had a teacher who shared a story like this: “I’m kind of deaf in one ear because I used to listen to music at such a high volume while cycling.” So, I thought, “Alright, I won’t do that.” I even have a feature on my phone that prevents me from going above a certain limit. But there are some songs where I add more bass because it feels better, or I increase the volume for better sound quality. But generally, I’m paranoid about it.

For all the participants, the use of hearing protection is specific to distinct situations, such as live concerts, whereas in other contexts, such as in school, they use headphones instead. The use of headphones in situations where one might traditionally use hearing protection may have consequences for hearing-health, particularly in settings where both the external noise level and the music in their headphones are played at high volumes. Another common experience is that the participants do not think that their peers wear hearing protection in general. The perceived lack of peer pressure contributes to a sense of freedom regarding whether to use hearing protection or not, as seen in the following quote:

It depends on the situation. If it’s just a regular school day that might get loud, I usually don’t bring [hearing protection] because I have music instead that blocks it out. But if I know in advance that it’s something really big where I think safety preparations will be needed, then I usually bring them. I don’t know anyone who usually wears hearing protection, so it’s always been a personal choice for me, whether to bring them or not; no one has said anything about it. So, it’s nice that there’s no peer pressure about it; it should be obvious that it’s different from person to person. (P2)

This exploration of music enjoyment and hearing-related risks illuminates the intricate relationship between the benefits experienced from (loud) music and the awareness of potential hearing-health consequences. The participants’ experiences emphasize a need for balanced and informed decision-making in order to continue enjoying music for a long time while preserving their hearing-health.

## 5. Discussion

### 5.1. Methodological considerations

Open-ended interviews inherently allow for a degree of flexibility and adaptability in data collection. While this is one of the strengths of the qualitative approach, enabling a more responsive interaction with the participants, it also introduces some challenges. There were variations in conversations with the participants, such as the line of questioning. This was addressed by ensuring that all question areas outlined in the interview guide were consistently covered with all the participants. Time was also spent critically reflecting on the interview technique to enhance the quality of the data and in turn the analysis. There is a potential risk of selection bias as adolescents who share an interest in music were intentionally included. This would have implications for the generalizability of the findings beyond this specific group and should be considered when interpretating the findings. However, the study also benefits from the inclusion of individuals who share an interest in music as it provides relevant in-depth insights which align with the aim of the study. Our sampling and recruitment strategy aimed to include a range of perspectives on music listening habits, while also ensuring that the selected participants were more than merely “regular” music listeners. Each participant, irrespective of their primary academic discipline, possesses a connection to music that exerts a great influence on their daily lives. Although the sample may seem highly varied due to the age range and different educational settings, the unifying factor among all participants is their significant engagement with music. This commonality aligns with the aim of the study, enables a deeper understanding of the impact of music across various listeners, and enhances the transferability of the findings to other similar contexts. The main strength of this study is the focus on the adolescents’ experiences with music, a population that has been overlooked in hearing health research until recent decades. By studying the motivations behind music listening among adolescents, the study fills an important gap in the literature. Furthermore, the qualitative approach emphasizes the lived experiences of the adolescents, which not only increases our understanding of their behaviours and attitudes towards music, but also informs the promotion of hearing health in this age group. Smith et al. (2022) define validity as the meaningfulness and trustworthiness of a study and emphasize that a focus on depth rather than breadth leads to higher quality. High-quality IPA studies aim to illuminate the participants’ experiences within and across cases. This includes highlighting how different participants manifest the same themes in unique ways. This study attempts to elucidate individual experiences, with the interpretative content contributing something more to the participants’ insights. This interpretive aspect, while effective in revealing nuanced insights, also introduces the possibility of personal biases which could potentially influence the findings and generalizability of the study (Smith et al., 2022). In this study, there has been an attempt to lessen the impact of personal biases through rigorous reflexivity and methodological checks. The narrative of the analysis moves between group-level claims, individual-level claims, and detailed analysis of the extracts used. The extracts used are rich, context-specific quotes that exemplify the findings derived from the analysis. Trustworthiness is ensured through a systematic documentation of the analysis process and by following the IPA method by Smith et al. (2009) and Smith et al. (2022). Co-judging and validity checking of the themes and interpretations of the data were implemented collaboratively to minimize subjectivity. The findings from the analysis also includes verbatim statements from the participants, allowing readers to interrogate our interpretations. Several checks on the analysis were conducted independently by co-authors, including a review of transcripts and inspection of emerging analytic content. The overall goal of the validity checks is not to seek a singular true account of the material, but rather to verify that the analysis presented has been systematically achieved and is supported by the data (Smith et al., 2022).

### 5.2. Main findings

The aim of this study was to investigate the role and meaning of music in adolescents’ lives through examining their lived experiences. Emphasis was placed on understanding how they perceive and understand music in relation to potential hearing-health risks. The findings reveal the many ways in which adolescents engage with music, highlighting its role as a daily companion that can enhance their well-being. Consistent with previous research on the meaning of music in adolescents’ lives, our findings corroborate that adolescents are aware of the influence of their musical choices. They purposefully use it to fulfil various important roles, such as mood and emotion regulation, creating inner worlds as a form of escapism, and shaping their perceptions of themselves and their environment (Beckmann, 2013; Groarke & Hogan, 2018; Miranda, 2013; Schäfer et al., 2013; Stewart et al., 2019; Upadhyay et al., 2017). The findings also raise questions about the perceived risks associated with music listening, particularly in relation to the enjoyment of loud music. A discussion will follow below, centring the meaning and roles of music, in relation to important factors to consider when trying to understand risky music listening.

#### 5.2.1. The risks and rewards of music listening

The findings indicate that music has an influence on emotional regulation, cognitive ability, and self-expression, this in turn influences how the participants understand potential hearing-health risks. Despite some acknowledgement of the potential risks associated with music listening, the participants often prioritize the benefits over potential long-term consequences, creating a tension between instant gratification and the preservation of hearing-health. In this context, music is seen as a valuable means of enhancing daily experiences and fostering positive emotions, and therefore not necessarily perceived as risk-taking. The primary motivation for listening to loud music is in the sensory experience it provides. Loud music improves the listening experience for the adolescents, creating a more immersive feeling both physically and emotionally. Additionally, they seem to consciously adjust the volume depending on specific settings and situations. This indicates an understanding of the impact that music can have on state of mind, thereby using it to achieve different goals and experience the various rewards involved, even if there is potential for harm in the long term.

While not consistently listening to what could be considered “loud” music, the descriptions of their music listening habits indicate continuous music listening during various daily tasks and situations. Though the volume may not be consistently high or the listening not attentive, there seems to be limited rest from auditory stimuli in this group. The absence of immediate discomfort or measurable hearing damage does not negate potential harm (Pienkowski, 2021; Putter-Katz et al., 2015). Previous research is not unanimous regarding the risks associated with volume and duration of music listening, as both factors are often identified as significant (e.g., Gilliver et al., 2017; Marron et al., 2014). All the participants exhibited a similar pattern of acknowledging the potential risks but not necessarily viewing their music listening as a form of risk-taking, or not being mindful of the risks in the moment. Lee and Jeong (2021) suggest that adolescents may not be actively conscious of the risks because potential consequences would manifest much later in their lives. Adolescents may not perceive loud music as an immediate threat to their health, and consequently, do not comprehend the severity. Therefore, information about the risks and consequences in early adolescence could be imperative (Jiang et al., 2016).

#### 5.2.2. Individual factors in music listening

The participants’ experiences reflect an inclination to engage in potentially risky music listening for emotion regulation. The immediate emotional benefits gained from music listening and loud music often take precedence over concerns about hearing-health risks. A systematic review found that several studies show that music can have positive effects on mood, well-being, and quality of life (Daykin et al., 2018). Our findings indicate that music can be used as a tool for improving moods and emotional states, which could partially explain why the risks associated with prolonged exposure to loud music may be underestimated. Similarly, Schäfer et al. (2013) found mood regulation as the most important function of music; motives linked to self-expression and mood regulation held significant importance, while those associated with social relatedness were less crucial. In addition, Garrido et al. (2022) explored how young individuals utilized emotion-regulation strategies while listening to music, such as matching their music to their mood to cope with sadness, anxiety, and fatigue. The emotional regulation aspect in music listening offers a deeper understanding of how music can be used for self-care. Based on our findings, the emotional benefits of music serve as a form of coping for the adolescents that leads to a positive health-related belief; they feel less at risk or vulnerable to health risks and negative consequences.

Music also plays a vital role in the formation of the adolescents’ identity (Chen et al., 2023). Through this stage of development, adolescents are exploring and shaping a sense of self; the music they prefer often aligns with their personal beliefs, morals, and how they see themselves. This process of self-discovery with music as a companion can have a substantial impact on the mental health and personal growth of adolescents (Chen et al., 2023). Our findings indicate that the participants’ music preferences are strongly linked to personal identity, more so than their music preferences serving as a tool for expressing their identity or forming a collective identity. The participants create their playlists based on their needs, mainly in relation to passing time and emotion regulation, their selection of music reflects an understanding of the personal benefits of music. Despite the potential for music to create relationships and establish connections within and between peer groups, it is important to acknowledge that not all adolescents use it intentionally for shaping social identity (Nuttall, 2009).

Our findings emphasize a preference for solitary music listening. The participants often use music and headphones specifically to isolate themselves from others and from external distracting noise. This finding exceeded initial expectations considering the common narrative that music often fosters social and communal relationships as well as to identify with youth culture (Sernhede, 1995). Our findings may be partially due to the increased social media interactions which may have influenced young people’s music listening habits. Many people prefer music listening through streaming services because smartphones and the internet are widely accessible and frequently used in many places (Kim et al., 2017). Free social media platforms are a fundamental part of music discovery and music sharing, streaming services provide easy access to global music libraries, encouraging exploration of various genres and artists (Lüders, 2020). The adolescents in this study are young inner-city inhabitants with high access to free social media and music streaming platforms. This context may play a role in shaping their music listening habits and potentially their broader cultural identities as peoples’ cultural identities can be influenced by digital interactions and media consumption (Humphrey & Bliuc, 2022). Social media influences how adolescents form and maintain relationships; fewer face-to-face interactions may alter adolescents’ approach to social interactions and exemplifies a broader social trend towards isolated, individualized experiences (Ball et al., 2023). This in combination with the preferred use of headphones for music listening can contribute to understanding the solitary music listening trend in our findings. It could be considered that the availability of such platforms is facilitating more frequent and varied music engagement. Future studies could investigate the extent to which digital access shapes music consumption patterns and explore how they differ in populations with varying levels of technological access.

Additionally, some research indicates that music listening preferences may vary as a function of individualist or collectivist cultures (Juslin et al., 2016). For example, collectivists may use music more often for dancing together with friends and family (Boer & Fischer, 2012). In collectivist contexts, it would be expected to see music experiences that are strongly tied to social circumstances (e.g., spending time with and dancing with family and friends (Boer & Fischer, 2012). However, in the individualist context, people may be more likely to seek out personal and emotional experiences (Juslin et al., 2016). When interpreting our findings about enjoying music as a private activity, it could be helpful to consider the cultural context of the study as western countries are often characterized by individualistic values (Humphrey & Bliuc, 2022). Therefore, it is reasonable to suggest that our findings are mainly relevant to similar contexts, and caution should be exercised when generalizing to different cultural or demographic groups.

In summary, changes in the lives of adolescents, driven by shifts in social and technological avenues, appear to influence their music preferences and listening habits. By considering these evolving dynamics and trends, the motivations behind and implications of potentially risky music listening among adolescents today can be better understood. Given the increased influence of social media, it may be effective to incorporate digital tools in hearing conservation interventions, to utilize social media for health promotion campaigns, and/or provide online resources that address the specific concerns related to music listening and hearing-health.

#### 5.2.3. Factors in risk-taking and decision-making

Social cognitive models have traditionally been used in health psychology research to explain health risk-taking and behaviour change towards a more health-oriented behaviour. The Theory of Planned Behaviour (TPB) (Ajzen, 1991) can contribute by emphasizing how social norms, attitudes, and intentions are important for explaining behaviour, while the Health Belief Model (HBM) (Rosenstock et al., 1988) highlights individual aspects such as perceived vulnerability and perceived barriers as crucial for behaviour change. Social cognitive theory (SCT), in addition, can offer insights into how individuals are influenced by their social environment and their own self-control mechanisms. With this study, the aim is to emphasize positive and personal aspects of music listening through adolescents’ experiences with music and how they understand potential associated health risks. These models can be used to relate music listening behaviour to risk-taking and behaviour change and will be discussed as part of interpretating the findings. However, these models can also be scrutinized as a way to enrich the findings. Our findings demonstrate that beyond the variables in these models, other key factors should be included when studying risk-taking in the context of music listening. This can help us understand potentially risky listening behaviour and, in the long run, what factors may influence a change in this behaviour.

One of our main findings is that there seems to be low awareness and limited knowledge about the potential risks to hearing health associated with consistent and/or loud music listening. This may in turn influence the ability to find a balance between enjoying music versus protecting one’s hearing. However, this does not necessarily mean that a lack of knowledge about long-term risks to hearing-health is a driving factor in decision-making. Emotional, personal, and habitual aspects seem to play a large role in the decision-making regarding the participants’ music listening habits. These are specific important factors which are not accounted for in traditional models of decision-making such as the HBM, which presumes that decisions are made on a rational level (Rosenstock, 1974); decisions are however often made based on emotions or former experiences. While these types of models can be valuable as a starting point to understand health behaviours, they require adaption or expansion with other relevant factors, such as the emotional benefits of loud music, to address the unique complexities of music listening behaviour among adolescents.

Another central aspect is the cognitive rewards experienced by the participants. Music is used as a companion in the background of daily life at home, in transportation and while being in public places or social settings. It creates a sense of time distortion (slowing down or passing faster, depending on the personal goal) and makes routine tasks easier and more enjoyable. This perspective emphasizes music as a coping mechanism for assuaging anxious or stressful thoughts, ultimately improving the overall quality of life. While several previous studies have also found that music can be helpful in making some people feel more productive and effective while working, studying, and reflecting (e.g., Chirico et al., 2015; MacDonald, 2013; Papinczak et al., 2015), the aspect of music as a constant background stimulus for the purpose of these specific benefits does not seem to be emphasized in hearing-related research. A more common approach includes the study of potentially beneficial or detrimental effects of music on attention and concentration (e.g., Burkhard et al., 2018; Cloutier et al., 2020). Consequently, this may lead to a limited understanding of why individuals engage in certain music listening behaviours and how it influences their state of mind. To make more informed decisions about health-related behaviours, it is important to consider these cognitive rewards alongside the factors in models such as the HBM or TPB.

Another main aspect from the findings concerns the adolescents’ reluctance to share their music preferences with their peers or in public. Typically, in the HBM, perceived susceptibility concerns the perceived vulnerability to be harmed as a result of a health risk behaviour (Rosenstock et al., 1988). However, for the adolescents in this study, vulnerability to be criticized for one’s music preferences seems to be an important factor. Consequently, the HBM could be further expanded to integrate the role of personal and social identity in shaping health behaviours. This could be used to explore how identity-related motivations (individualism or peer acceptance) impact perceptions of susceptibility and severity of health risks. There are similar shortcomings in other models which do not individually account for the relevant factors our findings have uncovered. For instance, according to the SCT, changes in the environment can result in changes within the individual, and although it emphasizes the interaction between individual, behaviour, and environment, the degree of influence by each factor remains unclear (Bandura, 1998; McAlister et al., 2008). More importantly in the context of this study, the SCT gives minimal attention to emotional and motivational aspects. Furthermore, the TPB does not incorporate factors that might play a significant role in shaping intention and drive either, such as desire or fear (Alhamad & Donyai, 2021). The personal and emotional connections to music can interact with risk awareness in complex ways. To better address risky music listening, models of decision-making would need to include factors that consider personal and emotional motivations which overshadow considerations of long-term health risks. This would involve identifying that health-related behaviours are perhaps not solely based on conscious assessment of risk and benefit. Instead, they seem to be influenced by how these behaviours contribute to emotional well-being.

There are also some practical factors appearing worth considering in relation to designing hearing prevention interventions. The participants recognize that recommended volume limitations on their smartphones is a useful function when listening to music, and they adhere to these limitations. It may be an effective strategy to implement a standard global volume limitation on all smartphones. Another important aspect is that they find it difficult to visualize how the act of increasing the volume may lead to a potential hearing loss in the distant future. While they have been told, for instance by parents or teachers, that listening to loud music for extended periods of time is generally considered to be bad for hearing-health, they express a want for more specific information about why it is harmful and how the consequences would personally affect them. Otherwise, the general advice can be easily dismissed. Furthermore, some of the participants have experienced tinnitus or ear pain after music listening and due to fear or anxiety of experiencing these symptoms again, they tend to lower the volume. Others have been influenced by these triggers simply by knowing someone who has experienced symptoms of hearing loss due to music listening. Therefore, it may be effective to create occasions for young people to discuss their perceptions of hearing-related risks with each other. This approach would limit the influence of authoritative figures, such as audiologists and parents, in decision-making, and instead highlight the prevalence of symptoms and the attitudes among their peers, potentially making the topic more relatable.

#### 5.2.4. Understanding risky music listening—changing the narrative

The findings from the thematic analysis show that music listening extends beyond mere entertainment or intrinsic and aesthetic enjoyment, revealing broader implications at play than hearing-related risks. It can be argued that it could also be risky for an individual’s mental health if they have ineffective emotion regulation (Menefee et al., 2022). Studies have shown that adolescence can be a particularly vulnerable time for the rise of mental health problems as well as for the development of emotion regulation (Helland et al., 2022). Thus, valuing the benefits of music does not only imply a disregard for hearing-health risks. Instead, it emphasizes the importance of using music as a tool to manage these other health risks effectively. Considering the participants’ experiences, music listening can create a space for the individual to exist without being judged or criticized, it can also be used for therapeutic purposes, allowing the regulation of state of mind, ultimately serving the purpose of improving emotional well-being. These benefits are perceived as a priority as opposed to considerations of potential long-term risks to hearing-health. Hence, risk-taking in music listening should not only be seen as an impulsive pursuit driven by sensation-seeking or lack of awareness. Rather, it seems more accurate to consider risky music listening behaviour as a consequence of the various benefits that music offers the individual. This can be important in the broader context of studying music listening habits and hearing-related risks as it offers a nuanced perspective and more balanced approach by acknowledging the benefits of music alongside the potential risks. This could lead to a more comprehensive understanding when forming recommendations for interventions and future research.

## 6. Implications for interventions and future research

Previous intervention studies have indicated that adolescents are more willing to engage in preventive behaviours, such as wearing hearing protection or reducing the frequency and duration of headphone use and volume, when they are made aware of the potential risks for permanent hearing loss (e.g., Gilliver et al., 2012; Taljaard et al., 2013). Research has also shown that educational campaigns, while leading to a modest increase in hearing protection usage at night clubs, had limited overall impact on behaviour change (Gilles & Paul, 2014; Weichbold & Zorowka, 2003, 2007). Vogel et al. (2010) found that it might be challenging to encourage voluntary behaviour change among adolescents, given that activities such as visiting live music venues, seemed to be deeply intertwined with the lifestyle of young people. Furthermore, a systematic review by Khan et al. (2018) found that there is limited evidence to support the efficacy of hearing conservation educational programmes aimed at young adults. This review also found that it may be more effective to conduct education hearing campaigns through web-based programs and smartphone apps (Khan et al., 2018).

Based on our findings, the positive effects of music listening seem to take precedence over potential health risks, as it may be easier to dismiss long-term effects compared to if they were immediate. If this Conclusion is correct for other samples of individuals in different studies, it can be vital for designing effective health promotion strategies tailored to this age group. This emphasizes the need for interventions that do not dismiss the positive implications of music, and that endorse sensory enjoyment while still protecting hearing-health. For example, educational information could focus on demonstrating methods to enjoy music safely without compromising on the intensity or energy (e.g., individually fitted hearing protection) that one may seek in loud music listening.

Future research should focus on creating integrative models that can investigate the complex interplay of cognitive, emotional, and socio-cultural factors in music listening behaviour better. Our findings indicate that it may be important for interventions in hearing loss prevention to function on an individual level, focusing on the aspects that most strongly influence personal experiences. There is also a need for longitudinal studies to understand how the identified factors, including personal and emotional, influence risk-taking over time, especially through the formative adolescent years into adulthood. Cross-cultural studies may also be useful to evaluate generalizability and may also have implications for theory development.

## 7. Conclusions

Our findings suggest that music plays a large and meaningful role in the daily lives and well-being of the adolescents. The findings further reveal diverse levels of awareness among the participants regarding the potential consequences of music listening, such as temporary or permanent hearing loss. The interplay between music enjoyment and risk awareness adds complexity to the understanding of adolescent music listening habits. The adolescents are aware of the risks associated with music listening, but often prioritize the perceived benefits to their well-being. There might also be a shift towards increasingly individualized music listening habits, driven by technological advancements, and increased social media interactions. This emphasizes the need for nuanced interventions and theoretical frameworks that reflect these multifaceted dynamics. Furthermore, current social cognitive models of decision-making do not fully capture all the relevant personal and environmental factors that may influence adolescents’ music listening behaviours. Effective interventions should address both the risks of loud music as well as its personal appeal to adolescents, including the positive implications. Future research should therefore focus on developing integrative models that consider personal, emotional, cognitive, and socio-cultural factors.

## Appendix B

***Interview Guide***

**Introduction**: Explain what the interview will be about/what is expected from the conversation, talk about the consent form, request signed consent.

**Background questions**

- Name, age, school (major/academic focus), parents’ educational background and occupation.

**Question area 1: Music listening habits**

- Share your experiences with music listening and how it is a part of your daily life in various situations. Possible prompt: In what ways do you listen, where/when/how often, and why in those specific situations?

**Question area 2: Effects of music listening**

- Talk about why you enjoy listening to music in your daily life.

**Question area 3: Attitudes**

- Talk about your thoughts on anything related to volume when listening to music. Possible prompts: What is important to you regarding the volume you listen to? Do you perceive any positive or negative aspects of high volume?

**Question area 4: Perceived behavioral control**

- Imagine a typical day/situation/activity when you usually listen to music and describe how you experience it compared to when you do not listen to music.
- Envision being in a public situation where you find the music/noise/volume uncomfortably loud, such as at a party, concert, motorsport event, amusement park, etc. What do you think you would do and why?

**Question area 5: Social aspects**

- Share your thoughts on music in social settings (e.g., when engaging with friends/family). Do you feel that music has any significance or impact on such a situation? Possible prompt: If there is no music in such a situation, do you miss it, and why?

**Question area 6: Meaning of music**

- Talk about what music means to you, what thoughts and feelings come up when you think about the meaning of music in your life?

**Question area 7: Identity/personality**

- Describe the type of music (for example genre) you prefer and why you think you are drawn to it. Possible prompt: If you like a particular song/artist, does it depend on something specific, such as genre, lyrics, a certain instrument, or something else?
- Share your thoughts on your music listening in regard to what your friends or others in society (e.g., via social media) listen to. Possible prompt: Are you influenced by what others listen to, if so, how?
- Talk about your music listening when you are alone compared to when you are with friends/family. Is there anything you like to listen to when you are alone compared to when you are with friends/family? If so, why? Possible prompt: Do you ever feel “embarrassed” to share your music interests with others?

**Question area 8: Hearing protection**

- Talk about your thoughts and experiences with hearing protection (e.g., earplugs). In what situations is it relevant for you to use hearing protection, and why?
- Describe if and how your use of hearing protection may or may not be influenced by others (friends). Possible prompt: Does your decision to use hearing protection depend on whether your friends use it and in what way?

**Question area 9: Risk**

- Talk about the risks you perceive with listening to music.
- Describe your thoughts about short-term versus long-term risks with loud music for your hearing. Possible prompt: Do you ever consider your hearing in relation to music listening today compared to the in the future?
- Talk about how you perceive your friends’ and family’s attitudes towards high volume/loud music listening compared to your own. Possible prompts: Do you and your friends/family talk about the risks of high volume? Do you think it is something that should be talked about?

**Question area 10: Ideal music experience**

- Describe how the ideal music experience would look like for you. What is the best way for you to listen to music and why?

**Conclusion**

- Is there anything else you would like to share about your music listening that we have not talked about yet?
